# Anti-titin antibody is associated with more frequent hospitalization to manage thymoma-associated myasthenia gravis

**DOI:** 10.3389/fneur.2022.978997

**Published:** 2022-10-05

**Authors:** Ki Hoon Kim, Seung Woo Kim, Jinhyuk Cho, Hye Yoon Chung, Ha Young Shin

**Affiliations:** ^1^Department of Neurology, Research Institute and Hospital of National Cancer Center, Goyang, South Korea; ^2^Yonsei University College of Medicine, Seoul, South Korea

**Keywords:** anti-titin antibody, myasthenia gravis, annual admission rate, thymoma, disease activity

## Abstract

**Background and purpose:**

Anti-titin antibodies are antistriational antibodies associated with thymoma-associated myasthenia gravis (MG). We evaluated whether the patients with anti-titin antibody are more frequently hospitalized to manage thymoma-associated MG than those patients without anti-titin antibody.

**Methods:**

Patients with thymoma-associated MG who conducted the serological test for anti-titin antibody were retrospectively included. Disease severity, treatments, MG-related annual hospitalization rate, and MG-related emergency room (ER) visit rate were compared between the patients with anti-titin antibody and those patients without anti-titin antibody. Multivariate analysis was conducted to analyze the association between anti-titin antibody serostatus and multiple admissions (hospitalization or ER visit of ≥2 times).

**Results:**

Of the 64 included patients, 31 (48.4%) patients were positive for anti-titin antibody (titin+ group) and 33 (51.6%) patients were negative for anti-titin antibody (titin– group). Both the annual rate of MG-related hospitalization and ER visit were significantly higher in the titin+ group [0.2 (0.1–0.6) and 0.1 (0–0.2) per year, respectively] than those in the titin– group [0 (0–0.2) and 0 (0–0) per year, *p* = 0.004 and *p* = 0.006, respectively]. In multivariate analysis, positive anti-titin antibody was still significantly associated with multiple admissions [odds ratio (OR) 4.11, 95% CI 1.05–16.03] compared to the titin– group as a reference after adjusting for sex, follow-up duration, age at onset, systemic chemotherapy, and the Masaoka staging.

**Conclusion:**

The presence of anti-titin antibody is associated with more frequent hospital utilization. Personalized explanation and careful monitoring strategy could be required in patients with thymoma-associated MG with anti-titin antibody for the timely detection of relapses.

## Introduction

Approximately, 85% of the patients with generalized myasthenia gravis (MG) have antibodies against antiacetylcholine receptor antibody (anti-AChR Ab). Besides anti-AChR Ab, several non-AChR autoantibodies have been identified in patients with MG. Some of these antibodies, termed striational antibodies, target epitopes on intracellular striated muscle proteins. Anti-titin antibodies are one of the striational antibodies, accounting for 20–30% of the patients with MG having anti-AChR Ab; these are more frequently present in thymoma-associated MG and late-onset MG (1–4). Titin is a large filamentous muscle protein that maintains the outline of the sarcomere and provides elasticity and flexibility to the sarcomere (5). As titin is an intracellular protein, it is unclear whether anti-titin antibody directly triggers an immune response in muscle fibers. However, it has been shown that the presence of anti-titin antibody is associated with coexisting nonneurological autoimmune disorders (6) or myositis (7). Thus, it can be assumed that anti-titin antibody has a possible link with various autoimmune conditions. Similarly, some of the previous studies demonstrated that anti-titin antibody positively correlated with disease severity in patients with MG (8–10).

Although a large number of studies have evaluated the association between anti-titin antibody and severity of MG (9, 11), not much has been elucidated on the aspect of clinical worsening of MG. Patients with MG frequently experience clinical worsening during the disease course. As frequent worsening can result in greater disability, fatigue, and poor quality of life, reducing the frequency of worsening is one of the clinical goals in managing MG. Even among the patients having low disease severity, patients with more frequent disease worsening could be considered as being uncontrolled. A recent study demonstrated that patients with anti-titin antibody were likely to receive combined immunosuppressive treatment and less likely to achieve remission (10), which may implicate that the patients with anti-titin antibody are experiencing frequent clinical worsening.

In this study, we hypothesized that the patients with thymoma-associated MG having anti-titin antibody may experience more frequent worsening with greater severity than those without anti-titin antibody. As the patients with MG experiencing significant clinical worsening tend to visit emergency room (ER) or require hospitalization, we compared the frequency of MG-related hospitalization and ER visit, as well as treatment status, between the patients with thymoma-associated MG with and without anti-titin antibody.

## Materials and methods

### Participants and data collection

We retrospectively reviewed the medical records of the patients diagnosed with thymoma-associated MG who visited the Department of Neurology at Severance Hospital between May 2017 and September 2020. Diagnosis of MG was made based on the presence of typical muscle weakness that worsens by fatigue, results of the repetitive nerve stimulation test, neostigmine challenge test, and serological tests for anti-AChR Ab. The patients with the following criteria were included: (1) diagnosis of MG, (2) histologic evidence of thymoma by thymectomy, and (3) who were tested for anti-titin antibody. Exclusion criteria were: (1) who were taking immunosuppressive treatment for an autoimmune disease other than MG, and (2) whose clinical data regarding onset and diagnosis of MG and disease severity was unavailable. Patients were classified into those with anti-titin antibody (titin + group) and without anti-titin antibody (titin– group).

Clinical information about age, sex, age at symptom onset, thymectomy, thymic pathology, thymoma recurrence, and laboratory findings was collected by reviewing medical records. Thymic pathology is classified based on the WHO Classification of Tumors of the Thymus (12). For the clinical staging of thymoma, the Masaoka staging system is used (13). The Myasthenia Gravis Foundation of America (MGFA) clinical classification was used to assess the clinical severity of MG at the point of the worst disease severity. The MGFA clinical classification ranges from class I (ocular muscle weakness only) to class V (state of intubation) (14). The Severance Hospital Institutional Review Board approved this study (Approval No. 4-2021-0002) and the study has been conducted in accordance with the Declaration of Helsinki.

### Anti-titin antibody

The presence of anti-titin antibody was determined by immunoblot analysis. Samples were tested by Euroimmun line blot assays (EUROLINE Neuronal Antigens Profile PLUS RST: amphiphysin, CV2, Ma2, Ri, Yo, Hu, recoverin, SOX1, and titin) according to the manufacturer's instructions. This consists of membrane strips separately coated with neuronal antigens, onconeural antigens, recoverin, SOX1, and titin. The result was interpreted with the EUROLineScan software in a binary fashion into positive and negative.

### Myasthenia gravis-related hospital visit

History of hospitalization and visit to ER for the management of MG were recorded by reviewing medical records from the time of diagnosis. Patients with follow-up duration of ≥ 1 year were selected for this analysis. MG-related annual hospitalization rate and MG-related ER visit rate were calculated by dividing the number of events by follow-up duration in year. Regular hospitalization for intravenous immunoglobulin (IVIg) maintenance therapy, hospitalization for elective thymectomy operation, and admission for other medical or surgical causes not related to MG were not included. Hospitalization or ER visit of ≥ 2 times was considered as multiple admissions.

### Statistical analysis

Data were expressed as number (percentage) for categorical variables, and as mean ± SD or as median (interquartile range) for continuous variables depending on compliance with the normal distribution. Clinical and laboratory data between the titin+ group and the titin– group were compared. The Pearson chi-square test or Fisher's exact test was used for categorical variables. Provided the data did not deviate from a normal distribution, we used the *t*-test for comparison between the two groups. The Mann–Whitney *U* test was used in case of data that were not normally distributed. The logistic regression analyses were used to estimate the association between the multiple admissions and clinical variables. Variables with *p* < 0.1 in the univariate analyses and sex were entered into the multivariate analysis to adjust confounding factors. Statistical analyses were performed using SPSS for Windows (version 25; SPSS, Chicago, Illinois, USA). Differences with a *p* < 0.05 were regarded as statistically significant.

## Results

### Patients

**Figure 1:**
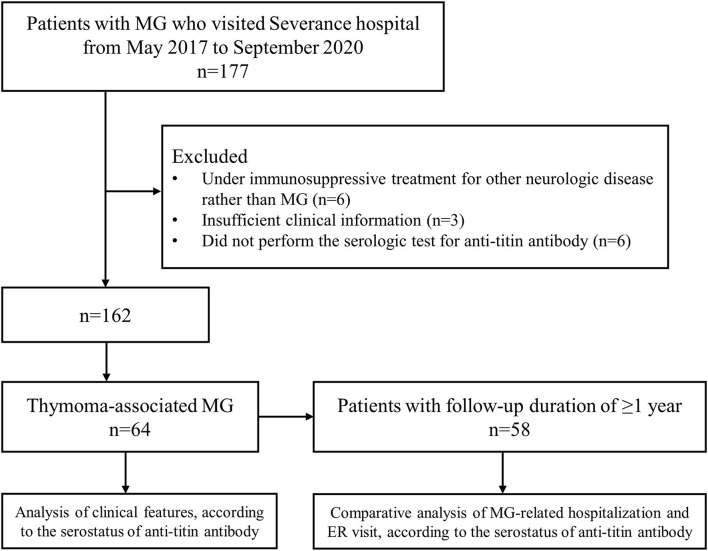
A flow diagram of patients. During the study period, 177 patients with MG visited hospital, and 64 patients who fulfilled the inclusion criteria were selected. MG: myasthenia gravis.

A total of 64 patients with thymoma-associated MG who underwent the serological test for anti-titin antibody were identified during the study period (Figure 1). Of these, 31 (48.4%) patients were positive for anti-titin antibody (titin+ group) and 33 (51.6%) patients were negative for anti-titin antibody (titin– group). The median duration from the onset of MG to the test for anti-titin antibodies was not significantly different between the titin+ group [74.4 Q1–Q3 (12.9–169.5) months] and the titin– group [50.4 (10.1–79.6) months, *p* = 0.169].

### Comparison of clinical features between the titin+ group and the titin– group

Clinical features are compared between the titin+ group and the titin– group as shown in Table 1. There was no significant difference in age at onset, sex, and anti-AChR Ab titer between two groups. Although the proportion of patients with the MGFA ≥ III was higher in the titin+ group (74.2%) than in the titin– group (57.6%), it was not statistically significant (*p* = 0.162). In terms of the treatment, the proportion of patients who had been treated with IVIg as rescue therapy was significantly higher in the titin+ group (45.2%) than in the titin– group (21.2%, *p* = 0.041). Patients under treatment with oral immunosuppressive agents tended to be higher in the titin+ group (74.2%) than in the titin– group (51.5%, *p* = 0.061). There was no significant difference between the two groups in thymoma pathology based on the WHO Classification and the Masaoka staging (*p* = 0.322 and *p* = 0.100, respectively). However, the proportion of patients with the Masaoka staging ≥ III was higher in the titin+ group (38.7 vs. 15.2%, *p* = 0.033). In addition, the rate of patients who received systemic chemotherapy was higher in the titin+ group (38.7%) than in the titin– group (6.1%, *p* = 0.002), as the proportion of patients who experienced thymoma recurrence (32.2 vs. 9.1%, *p* = 0.030).

**Table 1:** Comparison of demographic and clinical features in patients with thymoma-associated MG between the titin+ and titin– groups.

	**Titin+**	**Titin–**	***P*-value**
	**(*N* = 31)**	**(*N* = 33)**	
Age at onset, years	45.3 ± 11.3	46.7 ± 12.7	0.645
Duration from onset to titin test (month)	74.4 [12.9–169.5]	50.4 [10.1–79.6]	0.169
Sex, female	17 (54.8)	19 (57.6)	0.825
AChR antibody titer at diagnosis (nmol/L)	9.9 ± 4.8	9.8 ± 3.9	0.938
MGFA clinical classification at nadir			0.440
I	2	4	
II	6	10	
III	12	8	
IV	2	2	
V	9	9	
MGFA ≥III	23 (74.2)	19 (57.6)	0.162
MG crisis	9 (29.0)	9 (27.3)	0.876
Treatment–Rescue therapy			
IV methylprednisolone	13 (41.9)	9 (27.3)	0.217
IVIg	14 (45.2)	7 (21.2)	0.041
Plasma exchange	5 (16.1)	4 (12.1)	0.729
Number of rescue therapy	1 [0–2.5]	0 [0–1]	0.117
Treatment–Maintenance therapy			
Oral corticosteroids	29 (93.5)	27 (81.8)	0.259
Immunosuppressive agents	23 (74.2)	17 (51.5)	0.061
Regular IVIg	8 (25.8)	5 (15.2)	0.290
Thymoma pathology^*^			0.322
AB	2	7	
B1	2	5	
B2	11	13	
B3	7	5	
Masaoka staging^*^			0.100
I	2	5	
II	11	17	
III	4	3	
IV	8	2	
Masaoka staging≥III	12 (38.7)	5 (15.2)	0.033
Radiotherapy at anterior mediastinum	23 (74.2)	19 (57.6)	0.162
Systemic chemotherapy	12 (38.7)	2 (6.1)	0.002
Thymoma recurrence	10 (32.2)	3 (9.1)	0.030

MG, myasthenia gravis; MGFA, Myasthenia Gravis Foundation of America; MG, myasthenia gravis; AChR, acetylcholine receptor; IVIg, intravenous immunoglobulin.

Data are expressed as mean ± SD, median [interquartile range] or number (%).

* Of 64 patients who underwent thymectomy, pathological subtype of thymoma and the Masaoka staging was identified in 52 patients.

### Comparison of myasthenia gravis-related hospitalization and myasthenia gravis-related emergency room visit

The frequency of hospital utilization and treatment profiles were compared between the titin+ group (*n* = 29) and the titin– group (*n* = 29) who had follow-up duration of ≥1 year (Table 2). A total of 92 hospitalizations and 52 ER visits were observed during the study period. Main cause of MG-related admission was symptom aggravation (97.8%), followed by management of medication toxicity (2.2%). Main cause of MG-related ER visit was dyspnea or chest discomfort (61.5%), followed by limb weakness (19.2%) and bulbar symptoms (9.6%). In patients with more than 1 year of follow-up duration, both the time interval from symptom onset to serological test for anti-titin antibody and follow-up duration were not different between the two groups. The proportion of patients who experienced MG-related hospitalization or ER visit was significantly higher in the titin+ group (82.8 and 55.2%, respectively) than in the titin– group (48.3 and 20.7%, *p* = 0.006 and *p* = 0.007, respectively). Both the annual rate of MG-related hospitalization and ER visit were also significantly higher in the titin+ group [0.2 (0.1–0.6) and 0.1 (0–0.2) per year, respectively] than those in the titin– group [0 (0–0.2) and 0 (0–0) per year, *p* = 0.004 and *p* = 0.006, respectively].

**Table 2:** Comparison of MG-related hospitalization and MG-related emergency room visit between the titin+ and titin– groups among 58 patients^*^ with follow-up duration of ≥ 1 year.

	**Titin+**	**Titin–**	***P*-value**
	**(*N* = 29)**	**(*N* = 29)**	
Follow-up duration, year	5.1 [3.2–13.1]	4.8 [2.2–8.2]	0.331
Onset to anti-titin antibody test (month)	74.4 [17.9–169.1]	56.4 [24.4–106.2]	0.273
Onset to thymectomy (month)	4.0 [1.2–8.5]	3.1 [2.1–5.5]	0.658
MG-related hospitalization	24 (82.8)	14 (48.3)	0.006
MG-related ER visit	16 (55.2)	6 (20.7)	0.007
MG-related multiple admissions (≥2)	20 (69.0)	9 (31.0)	0.004
Frequency of MG-related hospitalization or ER visit			
Annual MG-related hospitalization rate	0.2 [0.1–0.6]	0.0 [0.0–0.2]	0.004
Annual MG-related ER visit rate	0.1 [0–0.2]	0.0 [0–0]	0.006
Duration of hospital stay per admission (day)	8.0 [5.0–15.5]	13.5 [6.0–21.3]	0.075

MG, myasthenia gravis; ER, emergency room; IS, immunosuppressant.

Data are expressed as mean ± SD, median [interquartile range] or number (%).

* Patients with short follow-up duration < 1 year were excluded.

### Association between multiple admissions and the presence of anti-titin antibody

Of 58 patients who had follow-up duration of ≥ 1 year, 29 (50.0%) patients had been hospitalized or visited ER more than once for the management of MG. The proportion of patients experiencing multiple admissions was significantly higher in the titin+ group than those in the titin– group (69.0 and 31.0%, *p* = 0.004). In univariate analysis, follow-up duration, high Masaoka staging (III or IV), systemic chemotherapy, and positive anti-titin antibody were associated with multiple admissions. In multivariate analysis, positive anti-titin antibody was still significantly associated with multiple admissions [odds ratio (OR) 4.11, 95% CI 1.05–16.03] compared to the titin– group as a reference after adjusting for sex, follow-up duration, age at onset, systemic chemotherapy, and the Masaoka staging (Table 3).

**Table 3:** Clinical variables associated with ≥ 2 times of MG-related hospitalization or ER visit.

	**Univariate**	**OR (95%CI)**	**Multivariate^+^**	**Adjusted OR (95% CI)**
	***P*-value**		***P*-value**	
Sex (female)	0.594	0.752 (0.264, 2.145)	0.228	0.429 (0.108, 1.700)
Age at onset	0.059	0.955 (0.910, 1.002)	0.109	0.952 (0.897, 1.011)
Onset to thymectomy	0.268	0.983 (0.953, 1.013)		
Anti-AChR antibody titer	0.287	1.066 (0.948, 1.199)		
Follow-up duration (year)	0.019	1.145 (1.023, 1.281)	0.023	1.164 (1.021, 1.326)
Thymoma recurrence	0.124	2.812 (0.754, 10.491)		
Systemic chemotherapy	0.021	5.296 (1.292, 21.715)	0.849	1.368 (0.055, 34.119)
Masaoka staging≥III	0.013	5.078 (1.406, 18.344)	0.441	2.926 (0.191, 44.807)
Positive anti-titin antibody	0.005	4.938 (1.623, 15.023)	0.042	4.111 (1.054, 16.033)

OR, odd ratio; CI, confidence interval; AChR, acetylcholine receptor.

+ Variables with p < 0.1 in the univariate analyses and sex were entered into the multivariate analysis.

## Discussion

Our results suggest that the patients with thymoma-associated MG having anti-titin antibody are more likely to experience more frequent hospitalization or ER visit to control MG than those without anti-titin antibody. In addition, the patients with thymoma-associated MG with anti-titin antibody were more likely to use IVIg and tended to use oral immunosuppressive agents more frequently than the patients without anti-titin antibody. The presence of anti-titin antibody could be utilized in predicting treatment response in patients with thymoma-associated MG.

Anti-titin antibody has been suggested to be a specific serological marker for MG (8). Especially, it had a tight association with thymoma-associated MG, which has been postulated that thymoma leads to altered presentation of autoantigen to T cells, resulting in intrathymic antigen-driven immune response (15, 16). Although its association with thymoma is well established (8), controversy remains in the association between the presence of anti-titin antibody and the severity of MG. Some of the previous studies showed that there is no significant association between the presence of anti-titin antibody and risk of myasthenia crisis, use of immunosuppressive agents, and disease severity (16, 17). In contrast, other studies have demonstrated that the presence of anti-titin antibody is associated with high disease severity, and changes in disease severity correlated with changes in titer of anti-titin antibody (8, 9). Hong et al. also showed that the patients with anti-titin antibody had worse modified Osserman classification, were likely to take combination immunotherapy, and were less likely to achieve favorable outcome (10). The result of the present study provides the association between anti-titin antibody and frequent clinical worsening.

The present study revealed that the patients with thymoma-associated MG having anti-titin antibody were more likely to be hospitalized or visit ER to manage MG. The annual frequency of hospitalization or ER visit related to MG was significantly higher in patients with anti-titin antibody than those without anti-titin antibody. This may implicate that the patients with both the thymoma and anti-titin antibody are likely to be uncontrolled or experience more frequent relapses, which are severe enough to require hospitalization or ER visit. Moreover, frequent relapses with hospitalization or ER visit profoundly affect the quality of life of the patients with MG. Relapse of MG, along with high disease severity and unresponsiveness to treatment, is associated with depression (18, 19), and the association between depression and worse quality of life has been consistently suggested in previous reports (20, 21). Other study showed that recent relapse of MG itself is associated with low quality of life (22). In addition, frequent hospitalization also imposes a considerable financial burden to the patients (23). Thus, careful assessment and monitoring strategy could be required in patients with thymoma-associated MG with anti-titin antibody to manage frequent relapses.

In the present study, anti-titin antibody was detected in 48.4% of the patients with thymoma-associated MG, which is slightly lower than those in previous reports, ranging from 50 to 76% (8, 10, 17, 24). This discrepancy is likely due to the difference in detection methods and timing of anti-titin antibody test. Anti-titin antibodies were mostly detected by the ELISA method with the MGT 30 domain in previous studies (8, 10), whereas the commercialized immunoblot kit was used in the present study. In addition, the median interval from disease onset to anti-titin antibody test was 62 months, which is much longer than those in previous studies (median or mean 17–32 months) (8, 10, 24). Given that levels of anti-titin antibodies were reduced according to the time interval after thymectomy (8), a long disease duration before the serological test could affect the seroprevalence of anti-titin antibodies in this study.

There are several shortcomings in this study. First, due to the retrospective nature of the study, the serological test for anti-titin antibody was conducted at different time points between the patients. In addition, a causal relationship between the serostatus of anti-titin antibody and hospitalization associated with MG cannot be determined based on the present study design. As the presence of anti-titin antibody is affected by various factors, including the severity of MG and whether the thymectomy is done (8, 9), interpretation of the serostatus of anti-titin antibody should be considered with caution. Second, the titer of anti-titin antibody was not evaluated in this study, and, thus, the association between the titer of anti-titin antibody and clinical outcomes cannot be determined. Third, hospitalization or ER visit could be affected by factors, including the personal preference, anxiety, economic condition, occupation, drug compliance, or distance between residence and hospital, which was not assessed in the present study. Finally, we were unable to provide the thymoma pathology in approximately one-fifth of patients due to the limited quality of medical records from other hospitals.

In conclusion, our results showed that the presence of anti-titin antibody has an association with more frequent hospital utilization in patients with thymoma-associated MG. Therefore, the serological test for anti-titin antibody can be used as a clinical indicator when treating patients with thymoma-associated MG.

## Data availability statement

The raw data supporting the conclusions of this article will be made available by the authors, without undue reservation.

## Ethics statement

The studies involving human participants were reviewed and approved by the Severance Hospital Institutional Review Board (Approval No 4-2021-0002). Written informed consent for participation was not required for this study in accordance with the national legislation and the institutional requirements.

## Author contributions

SK and HS: conceptualization and supervision. KK and JC: visualization. KK and SK: writing–original draft. All authors: resources and writing–review and editing. All authors contributed to the article and approved the submitted version.

## Funding

This study was supported by the National Research Foundation of Korea (NRF) grant funded by the Korean government (MSIT) (Grant No. NRF-2020R1C1C1010130).

## Conflict of interest

The authors declare that the research was conducted in the absence of any commercial or financial relationships that could be construed as a potential conflict of interest.

## Publisher's note

All claims expressed in this article are solely those of the authors and do not necessarily represent those of their affiliated organizations, or those of the publisher, the editors and the reviewers. Any product that may be evaluated in this article, or claim that may be made by its manufacturer, is not guaranteed or endorsed by the publisher.
